# Supplementary material for: On-surface synthesis platform for highly branched oligomers based on sequential C—C coupling and C—H activation of carbenes
Source: Nat Commun. 2026 Jan 30;17:1145. doi: 10.1038/s41467-025-67604-9 (PMC12858859; doi:10.1038/s41467-025-67604-9)
Supplement: Supplementary file 1 — Supplementary Information [file 41467_2025_67604_MOESM1_ESM.pdf]

Supplementary Information for

On-surface synthesis platform for highly branched oligomers based on sequential C−C coupling and C−H activation of carbenes

Yunjun Cao[1], Joel Mieres-Perez[2], Julien Frederic Rowen[3], Akshay Hemant Raut[3], Paul Schweer[1], Anran Bao[1], Wolfram Sander[3]*, Elsa Sanchez-Garcia[2]*, and Karina Morgenstern[1]*

[1]Physical Chemistry I, Ruhr-Universität Bochum, D-44801 Bochum, Germany

[2]Computational Bioengineering, Technische Universität Dortmund, D-44227 Dortmund, Germany

[3]Organic Chemistry II, Ruhr-Universität Bochum, D-44801 Bochum, Germany

*Corresponding authors: wolfram.sander@rub.de (W.S.); elsa.sanchez@tu-dortmund.de (E.S.G.); karina.morgenstern@rub.de (K.M.)

Contents

Supplementary Note 1: Assembly of precursor **1a** 2

Supplementary Note 2: IR spectra on Ag(111) 3

Supplementary Note 3: XP spectra on Ag(111) 5

Supplementary Note 4: Reactivity of carbene **2** 8

Supplementary Note 5: IET-induced vs. photolytic dissociation of precursor **1a** to carbene **2a** ... 9

Supplementary Note 6: Thermally-induced aggregation of carbene **2a** 11

Supplementary Note 7: Thermally-induced mixed chains from carbene **2a** and organometallic intermediate **3a** 12

Supplementary Note 8: Geometric flexibility of organometallic intermediate **3a** 13

Supplementary Note 9: Dimer **4a** from wet chemistry vs. on-surface synthesis 15

Supplementary Note 10: Bond-resolved STM images of substituted dimers **5a** to **10a** 16

Supplementary Note 11: IET manipulations for substituted dimers **5a** and **10a** 17

Supplementary Note 12: Formation and dimerization of carbene **2b** 19

Supplementary Note 13: C−H activation of carbene **2b** 20

Supplementary Note 14: Intramolecular cyclodehydrogenation 22

Supplementary Note 15: Synthesis of chemical compounds 24

Supplementary References 28

Supplementary Note 1: Assembly of precursor 1a

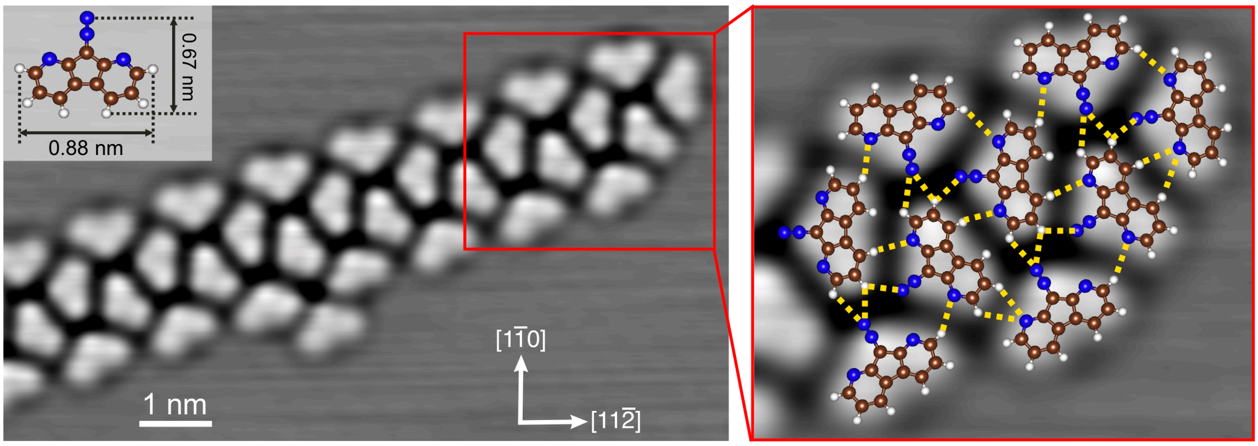

Supplementary Fig. 1. High-resolution STM image of ribbon pattern from precursor 1a with zoom superimposed with molecular models of **1a**. The image is processed by a Laplace filter. Scanning parameters: V_b = 10 mV, I_t = 1 nA. Yellow dashed lines mark the hydrogen bonds. Brown spheres: carbon atoms; blue spheres: nitrogen atoms; white spheres: hydrogen atoms. Inset: optimized geometry of **1a** on Ag(111). The Ag(111) surface is omitted for clarity.

Deposition of precursor **1a** at a low temperature of 50 K leads to a self-assembly into a ribbon pattern (Fig. 2 and Supplementary Fig. 1). In Laplace-filtered images, each molecule is imaged as a triangle with three protrusions, indicative of the two six-membered rings and the diazo group (inset of Supplementary Fig. 1). The superposition of the optimized geometries of **1a** reveals an ordered arrangement based on weak hydrogen bonds between the nitrogen atoms and hydrogen atoms of adjacent molecules (Supplementary Fig. 1, right).

Supplementary Note 2: IR spectra on Ag(111)

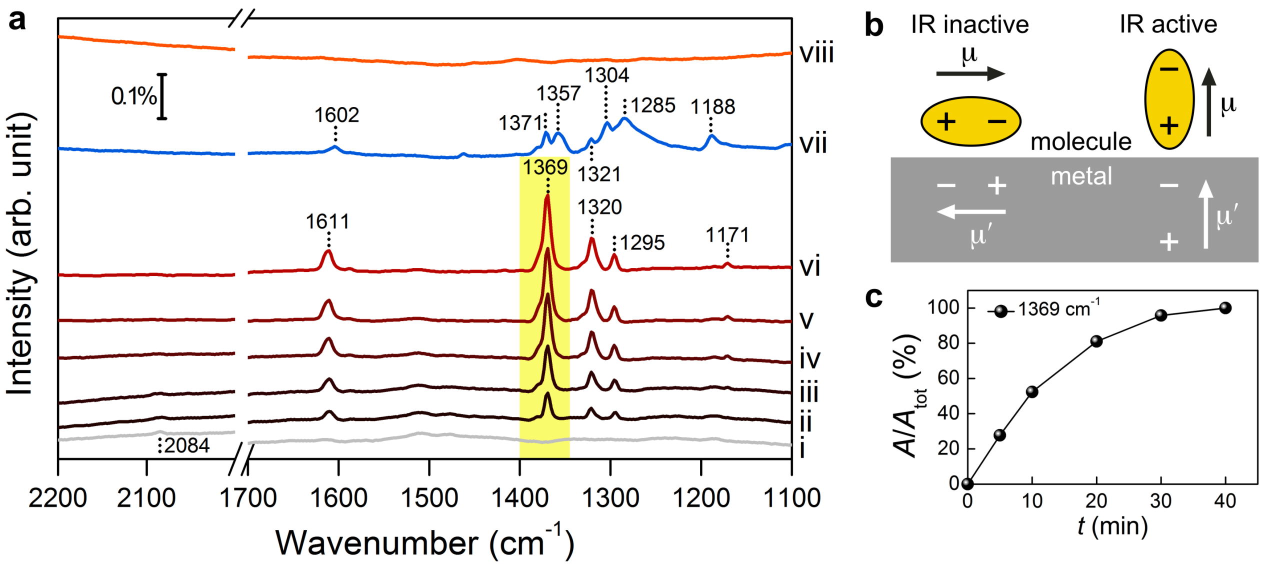

Supplementary Fig. 2. IR spectra of species on Ag(111). a, IR spectra after adsorption of **1a** on the Ag(111) surface at 105 K (i), after irradiation at 365 nm for 5 min (ii), 10 min (iii), 20 min (iv), 30 min (v), and 40 min (vi), and after annealing at 400 K (vii) and 600 K (viii). **b**, Scheme of the IR surface selection rule on metal surfaces. **c**, Relative intensities of the peaks (A/A_{tot}) marked in (b) vs. irradiation time (t). A and A_{tot} represent the peak areas at time t and at 40 min, respectively.

In the main text, we present DFT calculations that predict precursor **1a** and carbene **2a** having geometries parallel and tilted to the surface (Fig. 2). Here, we further corroborate these geometries by IR spectroscopy. Only a very weak peak at 2084 cm^{-1} is in the IR spectrum of precursor **1a** (curve i in Supplementary Fig. 2a), corresponding to an asymmetric C=N=N stretching mode. It is red-shifted by 12 cm^{-1} from its IR spectrum at 2096 cm^{-1} in argon matrices[1]. The low IR intensity for **1a** on the Ag(111) surface is explained by the IR surface selection rule[2]. On metal surfaces, a vibrational mode is IR active only if there is a non-vanishing projection of the dynamic dipole moment along the surface normal (Supplementary Fig. 2b). Thus, the IR spectrum supports the nearly parallel adsorption geometry of **1a** on the surface, as predicted by DFT calculations (Fig. 2f).

After irradiation, several strong IR peaks support the formation of carbene **2a** (curves ii to vi in Supplementary Fig. 2a). The peaks at 1611 cm^{-1} and 1171 cm^{-1} are close to the frequencies of the C−C−C bending and asymmetric stretching modes of **2a** in argon matrices, while the peaks at 1369 cm^{-1}, 1320 cm^{-1}, and 1295 cm^{-1} fall within the ranges of

the C−C stretching, C−N stretching, and C−H rocking modes. The dominant peak at 1369 cm^{-1} saturates in intensity after 40 min irradiation, indicating complete conversion of precursors **1a** to carbenes **2a** (Supplementary Fig. 2c). Notably, the 1369 cm^{-1} peak of carbene **2a** is approximately 26 times more intense than the 2084 cm^{-1} peak of precursor **1a**. Such a substantial increase in intensity suggests a change from a small to a large out-of-plane dipole moment component. It is explained by an adsorption geometry of **2a** with a large tilt angle, consistent with the geometry predicted by DFT calculations and the increased apparent height of **2a** relative to **1a** in STM experiments.

The peak shifts after annealing at 400 K (curve vii in Supplementary Fig. 2a) are consistent with the formation of organometallic dimer **3a**, reflecting a change in bonding environments. After annealing at 600 K, the IR spectrum is almost featureless (curve viii in Supplementary Fig. 2a). It suggests that the formed oligomers **5a** to **10a** are oriented nearly parallel to the surface, according to the IR surface selection rule.

The IR data clearly demonstrate the geometry change from **1a** to **2a**, as well as the subsequent reactions of **2a** upon annealing.

Supplementary Note 3: XP spectra on Ag(111)

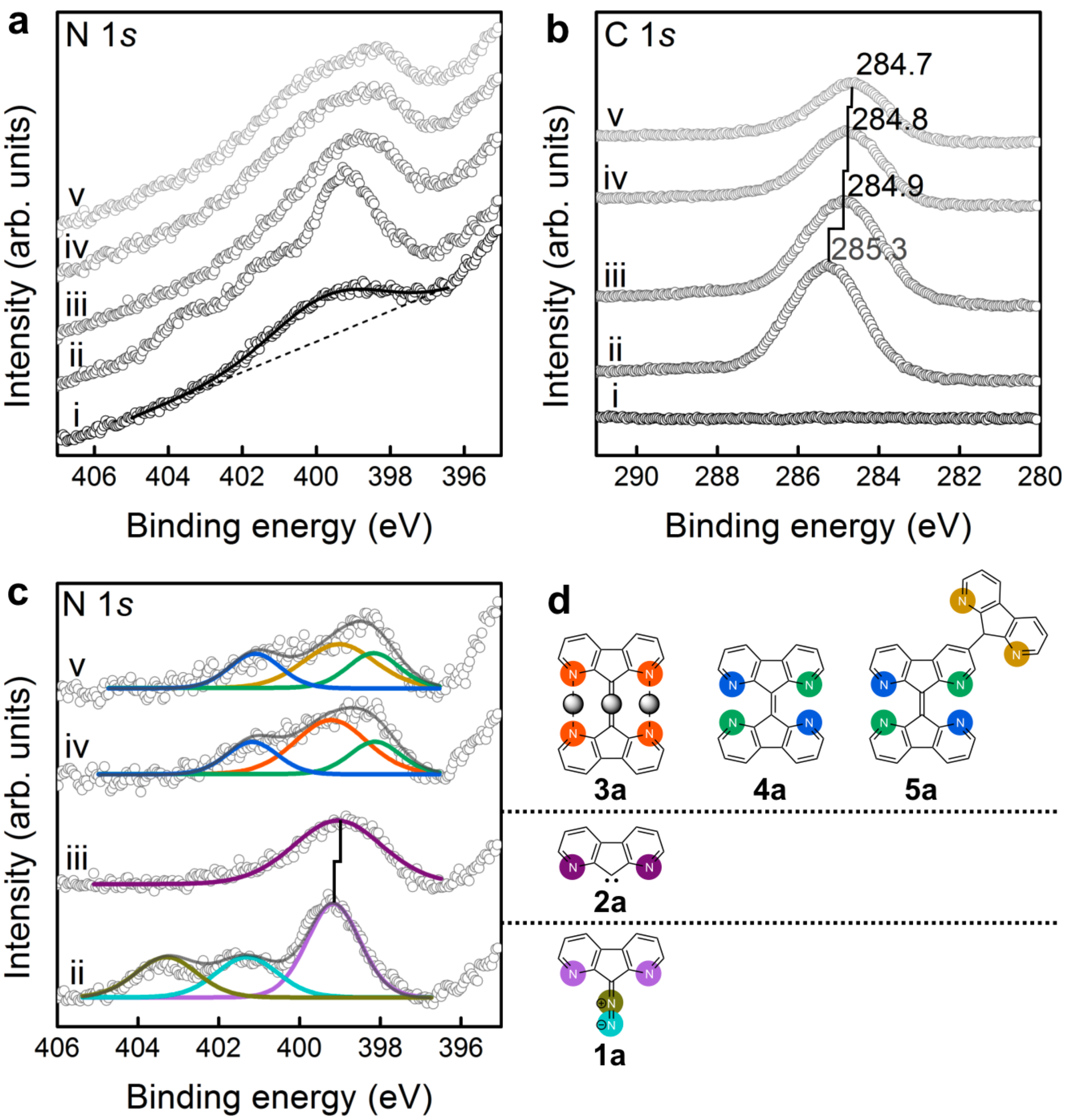

Supplementary Fig. 3. XP spectra on Ag(111). a,**b**, XP spectra of the N 1*s* and C 1*s* regions on a Ag(111) surface (i), after adsorption of **1a** at 87 K (ii), after irradiation at 365 nm for 2 h (iii), and after annealing at 400 K (iv) and 600 K (v). The black curve in (**a**) corresponds to a fit of the satellite peak of the Ag(111) surface on a linear background (dashed line). **c**, Background-subtracted N 1*s* spectra from (**a**) after subtracting the Ag(111) background (curve i in (**a**)), normalized to the areas of the related C 1*s* spectra in (**b**). For details, see Methods. **d**, Structural formulas of the reaction products with N atoms marked in the colors of the fits in (**c**).

In the main text, we studied the adsorption and dissociation of **1a** via STM (Fig. 2). Here, X-ray photoelectron spectroscopy (XPS) measurements are performed to obtain the

chemical identification of the species on the Ag(111) surface. XP spectra of the N 1*s* and C 1*s* regions are recorded for each reaction step (Supplementary Fig. 3a,b). To improve the visibility of the changes in the N 1*s* spectra, the surface-related satellite peak is subtracted, and the resulting spectra are normalized to the C 1*s* peak areas at this reaction step (Supplementary Fig. 3c; see Methods for details).

First, we confirm the non-dissociative adsorption of **1a** by XPS. The deposition of **1a** at 87 K yields three well-separated peaks with an area ratio of approx. 1:1:2, corresponding to the three chemical environments of the N atoms in **1a** (curve ii in Supplementary Fig. 3c). The area ratio suggests that the peak of higher intensity (light purple) corresponds to pyridinic N atoms within the molecular backbone[3]. Based on the N 1*s* spectrum of **1b** on the Ag(111) surface[4], the peaks at 403.3 eV (olive) and 401.3 eV (cyan) are assigned to the N atom binding to the C atom in **1a** and the terminal N atom, respectively. These results confirm that **1a** adsorbs non-dissociatively at 87 K, well above the maximum temperature of 52 K during irradiation.

Next, we confirm the activation of the C−N bond after irradiation and the evolution of the reaction intermediates at elevated temperatures. Upon irradiation at 365 nm, the 403.3 eV (olive) and 401.3 eV (cyan) peaks disappear, confirming the removal of the diazo group of **1a** (curve iii in Supplementary Fig. 3c). The byproduct is presumably molecular N_2, which desorbs at 87 K[5]. The removal of the diazo group is corroborated by a shift of the C 1s peak from 285.3 eV to 284.9 eV, suggesting the formation of **2a** (curve iii in Supplementary Fig. 3b). The pyridinic N peak shifts from 399.2 eV to 399.1 eV. In accordance with the disordered arrangement of **2a** observed by STM (Supplementary Fig. 4), the pyridinic N peak broadens after irradiation.

The pyridinic N peak splits upon annealing at 400 K (Supplementary Fig. 3c). According to the STM results, **3a** and **4a** form at this temperature (Fig. 3f). **3a** contains one type of N atom that is bonded via its lone pairs to an Ag adatom. The twisted geometry of **4a** produces two further types, but without bonding to an adatom, one closer to the surface and one farther away. Accordingly, the N 1*s* spectrum is fitted with three peaks yielding 399.2 eV

(orange) for **3a**, and 401.2 eV (blue) and 398.1 eV (green) for **4a**. The two outer peaks are assigned to the two nitrogens of **4a** because they persist in the next reaction step.

Upon annealing at 600 K, the N 1*s* peaks of **4a** remain, while the 399.2 eV peak of **3a** (orange) evolves into a 399.1 eV peak (dark yellow) assigned to the diaza-fluorenyl branches in the series of oligomers (Fig. 5a). The C 1*s* peak is shifted slightly to a lower binding energy (Supplementary Fig. 3b), from 284.9 eV (red) to 284.7 eV (olive). It is consistent with the formation of C−C bonds in the branched oligomers.

Overall, the XPS results support the reaction deduced from combining real-space imaging with DFT calculations.

Supplementary Note 4: Reactivity of carbene 2

The fundamental difference between carbenes **2** and the widely studied N-heterocyclic carbenes (NHCs)[6] is key to understand the surface reactivity of **2**. NHCs exhibit robust singlet states (S) with their lowest triplet states (T) lying 2 eV or more above the singlets[7]. Typical NHCs show high nucleophilicity and basicity but usually no electrophilic reactivity. In contrast, carbenes **2** exhibit triplet ground states in the gas phase with small (**2b**) to moderate (**2a**) S-T energy splitting[7]. In singlet carbene **2a**, the lone pairs at the N atoms strongly repel the lone pair at the carbene center, destabilizing the singlet state and increasing the singlet-triplet energy gap compared to **2b**[7]. The singlet states of **2** are even more nucleophilic and basic than typical NHCs, while simultaneously being extremely electrophilic. This combination of very high nucleophilicity and electrophilicity results in the unusual reactivity of **2**. It contrasts NHCs that are unreactive aside from acid–base reactions. While NHCs donate electron density to the surface[8], carbenes **2** act as electron acceptors[9]. Despite the very strong binding of carbenes **2** to metal surfaces, they maintain most of their reactivity[10], which makes them ideal building blocks for on-surface syntheses.

Supplementary Note 5: IET-induced vs. photolytic dissociation of precursor 1a to carbene 2a

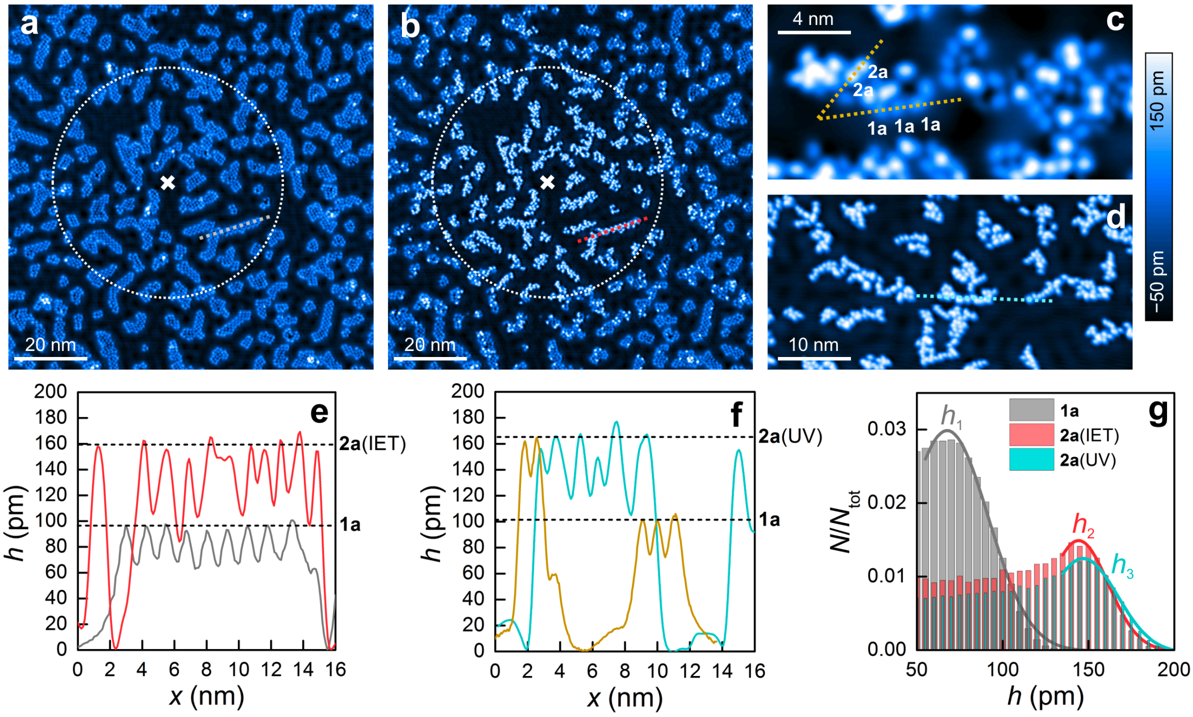

Supplementary Fig. 4. IET-induced vs. photolytic dissociation of precursor 1a to carbene 2a. **a**,**b**, STM images before (**a**) and after (**b**) indirect IET manipulation at the white cross. Manipulation parameters: V_b = 2.0 V, I_t = 105 pA, and t = 10 s. White circles mark the primary region of the dissociation events to guide the eye. **c**,**d**, STM images after irradiation of **1a** at 365 nm for 2 h (**c**) and 3 h (**d**). The STM tip is retracted during irradiation such that different regions of the sample are imaged. **e**,**f**, Apparent height profiles along the lines in (**a**) to (**d**). **g**, Pixel histograms of apparent heights normalized to the total number of pixels (N_{tot}) with Gaussian fits. Each histogram represents a statistics of approximately 1000 molecules. The fitted peak positions are h_1 = (68 ± 1) pm, h_2 = (144 ± 1) pm, and h_3 = (147 ± 1) pm. The surface baselines are set to 0 pm. Scanning parameters: V_b = 50 mV, and (**a**,**b**) I_t = 5 pA, (**c**,**d**) 10 pA.

In the main text, we generated individual carbenes **2a** from precursors **1a** through direct IET manipulation at a voltage of 1.0 V (Fig. 2). Additionally, indirect IET manipulation[11] leads at a higher voltage of 2.0 V to the same result in a larger surface region. It is evidenced by the increased apparent height of the formed species (Supplementary Fig. 4a,b). Still, indirect IET manipulation leads to a limited number of carbenes **2a** (Supplementary Fig. 4a,b). We employ UV irradiation to form carbene **2a** across the entire Ag(111) surface (Supplementary Fig. 4c,d). For this aim, we irradiate precursor **1a** on Ag(111) at 365 nm,

the same wavelength used for the dissociation of 9-diazofluorene to carbene **2b** on the same surface[9]. After 2 h of irradiation, some of the precursors **1a** have dissociated (Supplementary Fig. 4c). By 3 h, all precursors **1a** have dissociated (Fig. 4d). The photo-induced species is identical to the species induced by direct and indirect IET manipulation (Fig. 2 and Supplementary Fig. 4b), as confirmed by the same apparent height increase relative to precursor **1a**. IET- and photo-induced **2a** increase in apparent height by (62 ± 7) pm and (68 ± 9) pm (Supplementary Fig. 4e,f). Pixel histograms of IET- and photo-induced **2a** increase by (76 ± 1) pm and (79 ± 1) pm (Supplementary Fig. 4g). Thus, precursor **1a** is photolytically dissociated to carbene **2a** on the entire Ag(111) surface.

Supplementary Note 6: Thermally-induced aggregation of carbene 2a

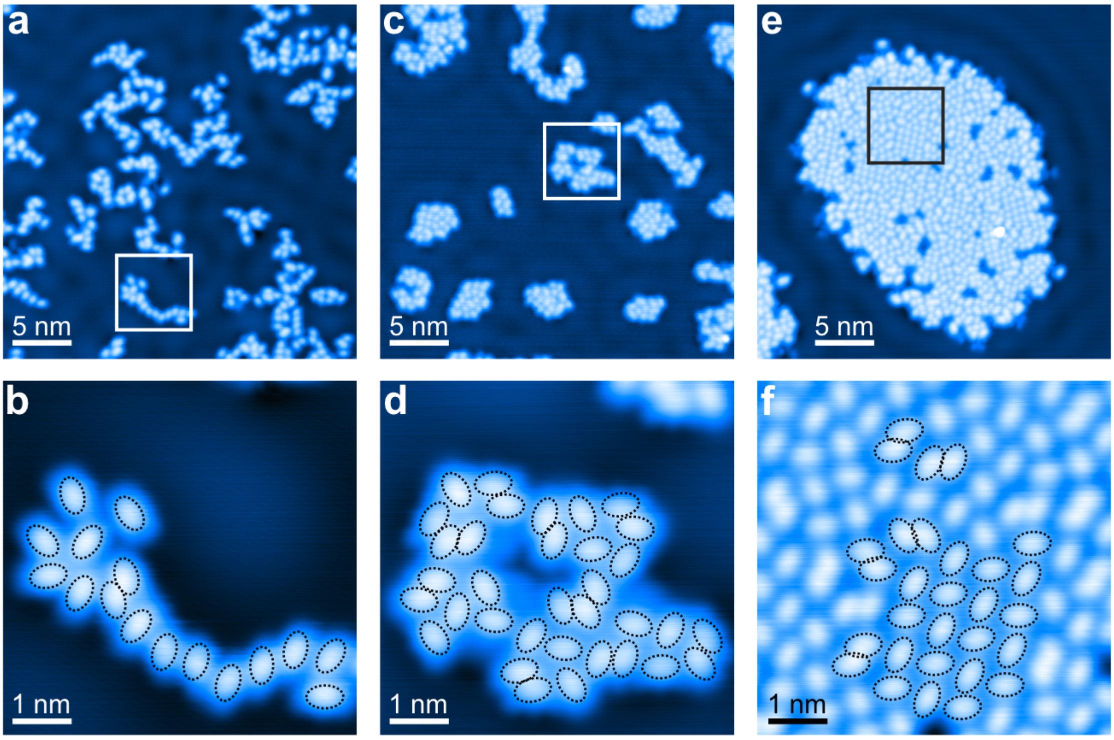

Supplementary Fig. 5. Thermally-induced aggregation of carbene 2a. a-f, STM images of carbene **2a** from photolysis of precursor **1a** at 52 K (**a**,**b**) and after subsequent annealing at 180 K (**c**,**d**) and 240 K (**e**,**f**). (**b**,**d**,**f**) Magnified images of the squares in (**a**,**c**,**e**). Dashed ellipses mark individual carbenes **2a**. Scanning parameters: (**a**,**e**) V_b = 50 mV, (**b**,**d**) V_b = 10 mV, (**c**) V_b = 100 mV, (**f**) V_b = 5 mV, and (**a**,**c**) I_t = 5 pA, (**b**,**d**,**f**) I_t = 100 pA, (**e**) I_t = 10 pA.

In Supplementary Note 5, we demonstrated that IET manipulation (5 K) and UV irradiation (< 52 K) dissociate precursor **1a** to carbene **2a** on the Ag(111) surface. In this section, we reveal a mobility of carbene **2a** during annealing. Other than precursor **1a** that self-assembles into ordered ribbons (Supplementary Fig. 4a), the carbene **2a** aggregates into clusters without long-range order (Supplementary Fig. 5a,b). Upon annealing at 180 K and 240 K, the unordered clusters condense into smaller (Supplementary Fig. 5c,d) and larger (Supplementary Fig. 5e,f) clusters with small domains of checkboard order. It confirms that carbene **2a** is highly mobile during reactions performed above 240 K. The thermally-induced aggregation of carbenes **2a** is crucial as it facilitates a proximity of carbene molecules that is essential for their coupling, demonstrated in the main text (Fig. 3).

Supplementary Note 7: Thermally-induced mixed chains from carbene 2a and organometallic intermediate 3a

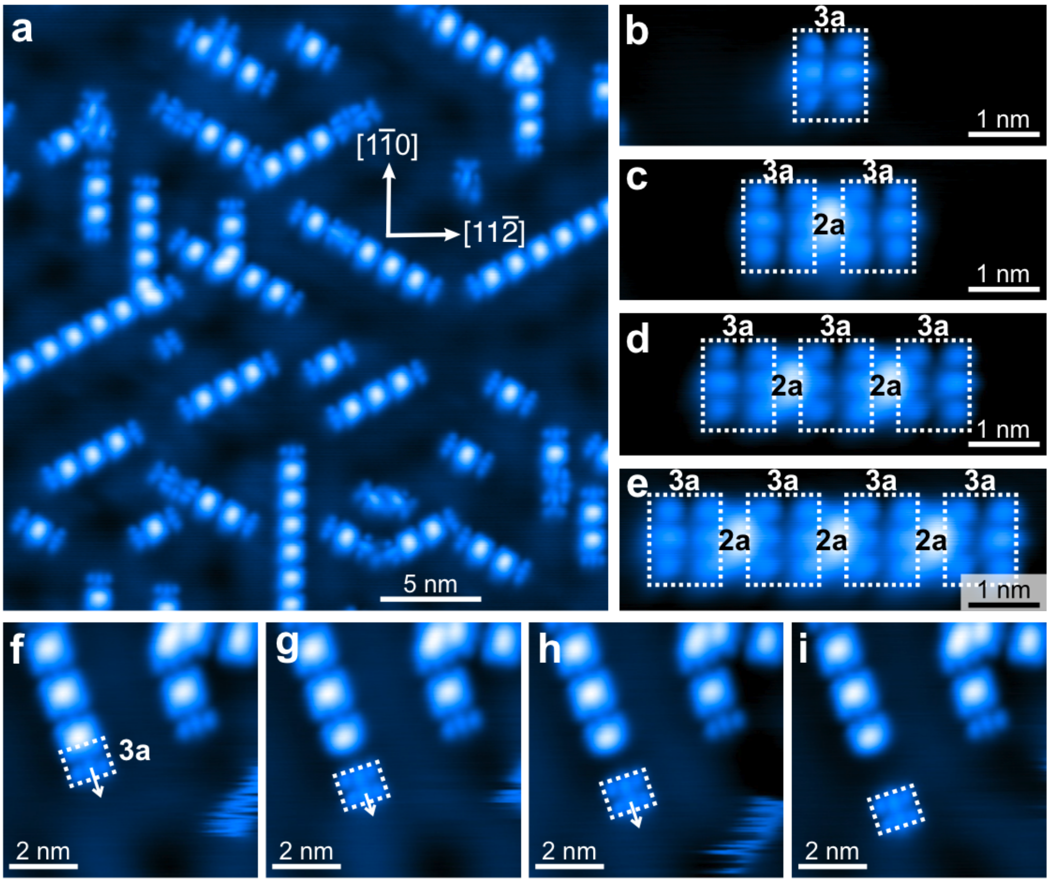

Supplementary Fig. 6. Thermally-induced mixed chains from carbene 2a and organometallic intermediate 3a. a, Large-scale STM image after annealing carbene **2a** on Ag(111) at 292 K. **b-e**, Small-scale STM images of a series of chains of increasing lengths. The images are rotated to align the chains to each other. Dashed rectangles mark organometallic intermediates **3a**. **f-i**, STM image series of lateral manipulations in constant-current mode along the white arrows at a tunneling resistance of 125 kΩ. Scanning parameters: (**a**) V_b = 50 mV, (**b-i**) V_b = 10 mV, and (**a**, **f-i**) I_t = 10 pA, (**b-e**) I_t = 1 nA.

Annealing the ordered carbene **2a** islands leads to chain structures (Fig. 3c and Supplementary Fig. 6a). These chains are characterized by an alternating pattern of six-protrusion species (dashed rectangles in Supplementary Fig. 6b-e) and ellipsoidal bright protrusions. Each chain terminates at both ends with the six-protrusion species.

Through lateral manipulation, we separate a six-protrusion species from a chain (Supplementary Fig. 6f to g). It is identical to an isolated six-protrusion species (Supplementary Fig. 6b). The six-protrusion species is moved as a whole during

subsequent manipulations, suggesting a stronger bonding of its parts within the species than to the surface (Supplementary Fig. 6g to i). The size of a carbene fits favorably to three protrusions, suggesting that the six-protrusion species consists of two carbenes. Based on the distance between the two pairs of three protrusions each, we propose that the six-protrusion species is a carbene-metal complex with two carbene molecules linked by three Ag adatoms (cf., organometallic intermediate **3a** in Fig. 1). The lone pairs of the nitrogen atoms interact with the two outer Ag adatoms. The two carbene centers interact with the central Ag adatom. Such a geometry is in agreement with the DFT calculations and fits favorably to the STM image of the main manuscript (Fig. 3i,j).

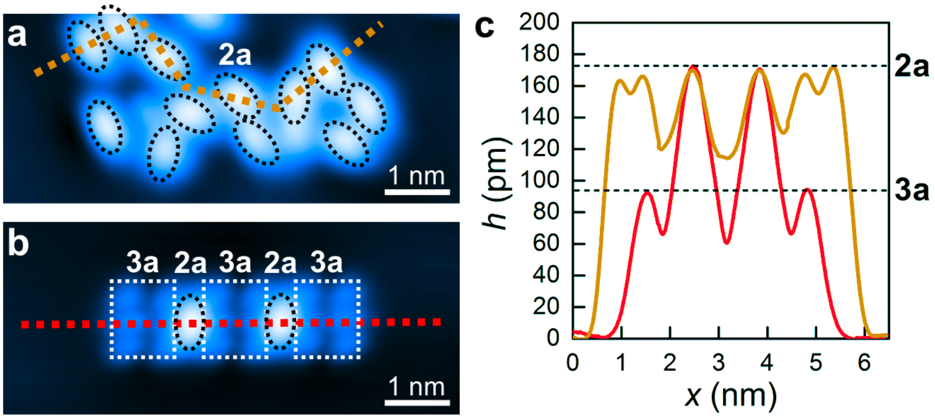

Supplementary Fig. 7. Carbene 2a in an aggregate and a chain. a, STM image of carbene **2a** from photolysis of precursor **1a** at 52 K. **b**, STM image of a short chain after annealing the photo-induced carbene **2a** at 292 K. Dashed ellipses and rectangles mark carbene **2a** and organometallic intermediates **3a**. **c**, Apparent height profiles along the lines in (**a**) and (**b**) in the corresponding colors. Scanning parameters: V_b = 10 mV, and (**a**) I_t = 10 pA, (**b**) I_t = 100 pA.

The size of the ellipsoidal bright protrusions between the six-protrusion species (Supplementary Fig. 7b) is similar to that of the carbene **2a** from photolysis of precursor **1a** at 52 K (Supplementary Fig. 7a). Moreover, they have a similar apparent height at the same bias voltage of 10 mV (Supplementary Fig. 7c). Therefore, we assign the ellipsoidal bright protrusions to unreacted carbenes. Annealing at a higher temperature of 375 K converts these ellipsoidal bright protrusions to organometallic intermediates **3a** (Fig. 3h), a strong indication that unreacted carbene **2a** was present before this annealing step.

Supplementary Note 8: Geometric flexibility of organometallic intermediate 3a

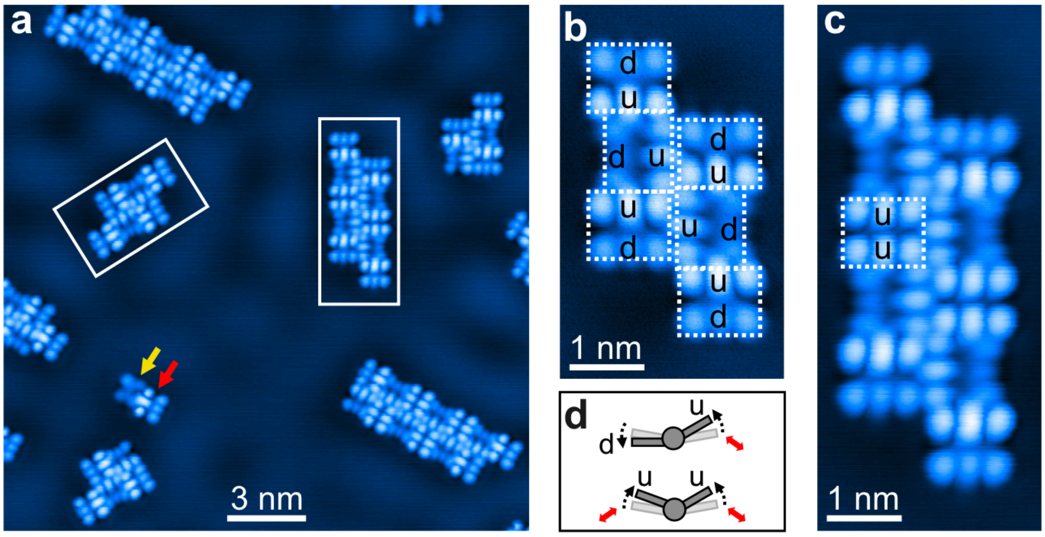

Supplementary Fig. 8. Geometric flexibility of organometallic intermediate 3a. a, STM overview image after annealing **2a** on Ag(111) at 375 K. **b**,**c**, Magnified STM images of the rectangles in (**a**). Dashed rectangles mark the contours of dimer **3a**, with u and d denoting the parts of dimer **3a** that are farther from and closer to the surface, respectively. Scanning parameters: V_b = 10 mV, and (**a**,**c**) I_t = 5 pA, (**b**) I_t = 1 nA. (**d**) Schematic of the geometry changes of dimer **3** due to intermolecular interactions (red arrows) with adjacent molecules.

In the main text, the calculated geometry of organometallic intermediate **3a** shows slight asymmetry (Fig. 3j). It is reflected in the geometric flexibility of the organometallic intermediate **3a**. While an isolated organometallic intermediate **3a** is more symmetric (Fig. 3e), the structure of a non-isolated **3a** is influenced by neighboring molecules. For instance, the three protrusions close to **4a** (yellow arrow in Supplementary Fig. 8a) are imaged brighter than the three protrusions away from **4a**.

In addition, the assembly of organometallic intermediate **3a** also shows asymmetry (u and d in Supplementary Fig. 8b,c). In such an assembly, each dimer is rotated 90° relative to its neighbors. Supplementary Fig. 8d illustrates the possible geometrical changes due to intermolecular interactions. When organometallic intermediate **3** interacts with an adjacent molecule only on one side, that side is farther away from the surface (u) while the opposite side is closer to it (d). If both sides of organometallic intermediate **3** interact with adjacent molecules, it adopts a geometry where both sides are farther away from the surface (uu).

Supplementary Note 9: Dimer 4a from wet chemistry vs. on-surface synthesis

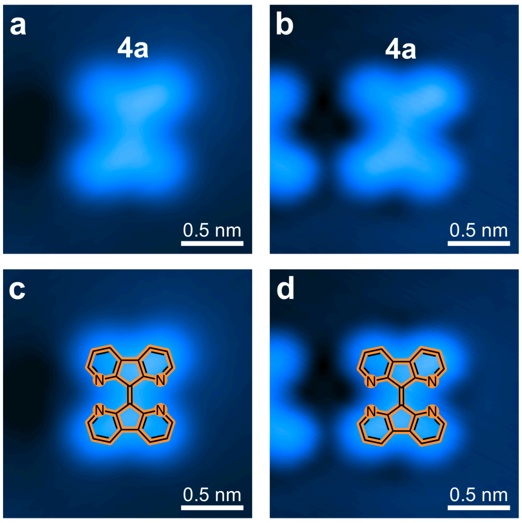

Supplementary Fig. 9. Dimer 4a from wet chemistry vs. on-surface synthesis. a-d, STM images of dimer **4** from (**a**) wet chemistry and (**b**) on-surface synthesis, with superimposed molecular models in (**c**) and (**d**). Scanning parameters: V_b = 50 mV, and (**a**,**c**) I_t = 1 nA, (**b**,**d**) I_t = 5 pA.

In the main text, we assigned the butterfly-shaped species formed after annealing at 375 K to dimer **4a** (Fig. 3). Dimer **4a** (Supplementary Fig. 9a) results from the C−C coupling of two carbenes **2a**. To verify this assignment, we synthesize dimer **4a** by a wet-chemistry approach (see Supplementary Note 15) and deposit it on the Ag(111) surface at 140 K. Indeed, the high-resolution STM image of dimer **4a** (Supplementary Fig. 9a) echoes that of dimer **4a** from annealing carbene **2a** at 375 K (Supplementary Fig. 9b). Both species are characterized by a butterfly shape composed of two fused ellipsoids, a shape that fits perfectly to the molecular models of dimer **4a** (Supplementary Fig. 9c,d).

Supplementary Note 10: Bond-resolved STM images of substituted dimers 5a to 10a

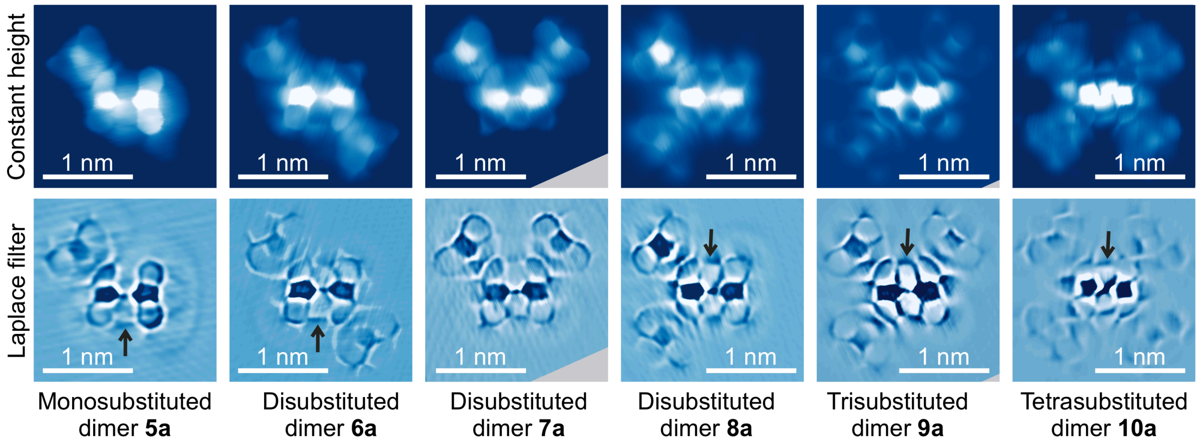

Supplementary Fig. 10. Bond-resolved STM images of substituted dimers 5a to 10a. Original STM images of substituted dimers **5a** to **10a** recorded with a functionalized tip in constant-height mode (upper row), and their corresponding images processed with a Laplace filter (bottom row). The images are rotated or mirrored to align the central dimer **4a**. For arrows: see text. Scanning parameters: V_b = 5 mV and I_t = 20 pA.

In the main text, bond-resolved STM images recorded with a functionalized tip confirm the formation of substituted dimers **5a** to **10a** (Fig. 4). Some lines (marked by arrows in Fig. 4 and Supplementary Fig. 10) are imaging artifacts, which are well-known in high-resolution AFM/STM images. These artifacts arise from the bending of the probe molecule on the tip[12] and appear between closely-spaced adjacent atoms that do not bond[13].

Supplementary Note 11: IET manipulations for substituted dimers 5a and 10a

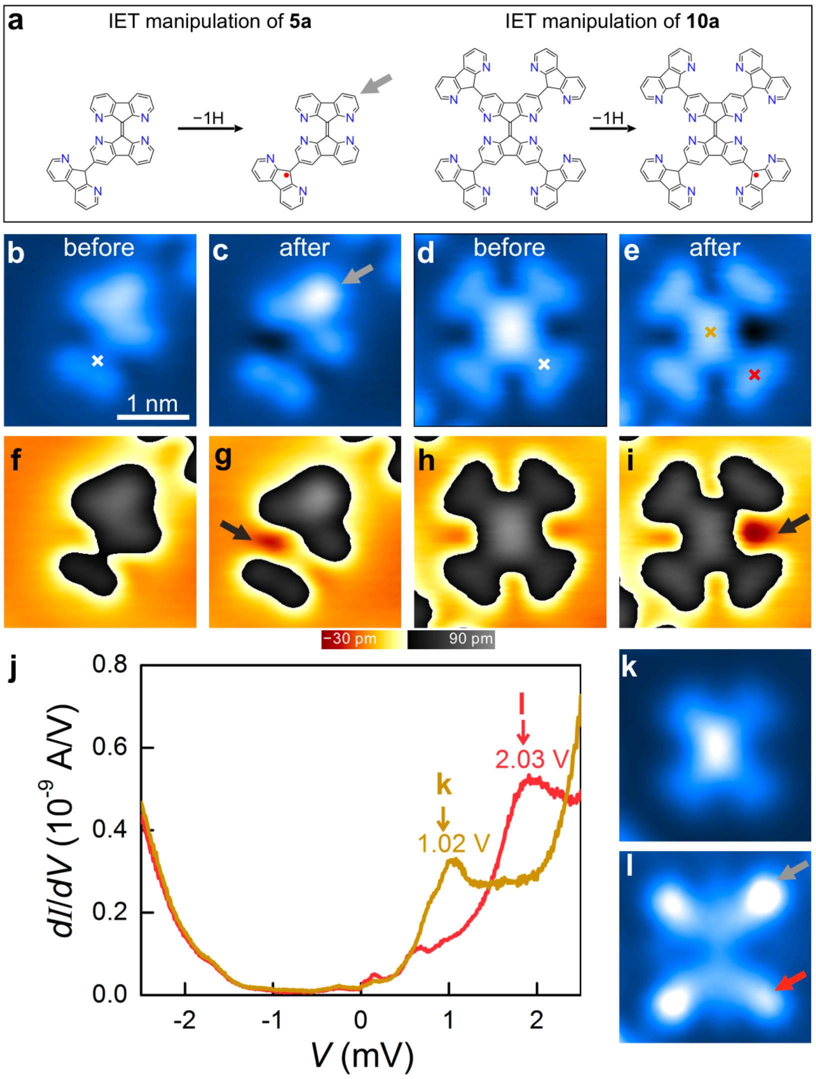

Supplementary Fig. 11. a, Scheme of IET manipulations for **5a** and **10a**. **b-e**, STM images of substituted dimers **5a** (**b** to **c**) and **10a** (**d** to **e**) before and after *I*-*V* manipulation up to 2.5 V at the white crosses. **f-i**, STM images in (**b-e**) are displayed with a double-color scale to enhance the contrast. Scanning parameters: V_b = 5 mV, I_t = 20 pA. **j**, dI/dV spectra recorded at the crosses in (**e**) in corresponding colors. **k**,**l**, dI/dV maps recorded at 1.02 V (**k**) and 2.03 V (**l**) with a tunneling current of 100 pA, a modulation voltage of 20 mV, and a modulation frequency of 413.3 Hz. All images use the same length scale.

As discussed in the main text, the carbene C−H insertion in solution leads to sp^3 carbon atoms via a concerted process involving C−C bond formation and hydrogen atom migration[14]. To confirm whether or not the sp^3 carbon atoms persist on the surface, we performed IET manipulations at the carbon of interest and monitored the induced changes using STM/STS and dI/dV mapping. We expected that a hydrogen can be removed from

an *sp*^{3} carbon site in the range of 2 eV to 3 eV[15,16]. A similar manipulation at a dehydrogenated sp^2 site should not alter the molecule[17].

For substituted dimers **5a** and **10a** (Supplementary Fig. 11a), the manipulations clearly alter the molecules (Supplementary Figs. 11b-f). The adjacent depressions in the subsequent STM images (Supplementary Figs. 11g, i) indicate a charge transfer from the molecule to the surface[18], consistent with the conversion from an *sp*^{3} to an *sp*^{2} carbon atom. For **5a**, one corner becomes brighter, most likely due to a tilting of the pyridine ring (gray arrows in Supplementary Fig. 11a and 11c). It can be caused by a reduced molecule–surface distance at the newly formed *sp*^{2} carbon site. For **10a**, a similar topographic change is quenched by a diaza-fluorenyl group at the same position. Therefore, we performed d*I*/d*V* spectroscopy and d*I*/d*V* mapping to monitor the electronic states of **10a** after manipulation. The spectra show two unoccupied molecular states at 1.02 V and 2.03 V at the dimer and the manipulated part of the molecule, respectively (Supplementary Fig. 11j). The d*I*/d*V* map at 1.02 V is dominated by a symmetric protrusion at the core of the molecule (Supplementary Fig. 11k). In contrast, in the dI/dV map at 2.03 V the C2 symmetry is broken and the local density of states at the manipulation site is reduced (red arrow in Supplementary Fig. 11l). The neighboring branch is brighter (gray arrow in Supplementary Fig. 11l), likely due to a geometry change. No Kondo resonance is observed in the d*I*/d*V* spectra, which is consistent with the quenched magnetism caused by the charge transfer.

In summary, the site-specific manipulation, the characteristic manipulation voltages (< 3 V), and the correlated topographic and electronic changes support the presence of sp^3 carbon atoms in the substituted dimers.

Supplementary Note 12: Formation and dimerization of carbene 2b

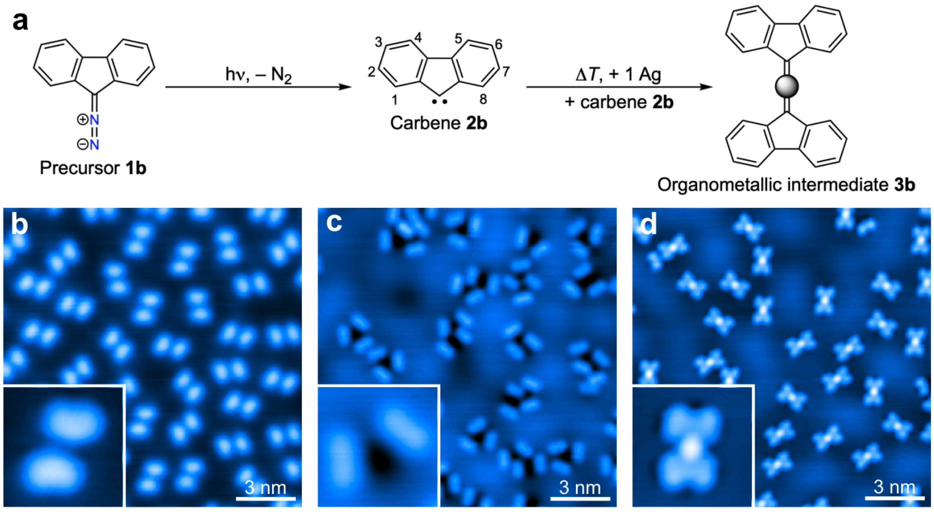

Supplementary Fig. 12. Formation and dimerization of carbene 2b. a, Scheme of the photolysis of precursor **1b** and the thermally induced dimerization of carbene **2b**. **b-d**, STM images of (**b**) precursor **1b** deposited on Ag(111) at 85 K, (**c**) after 23.2 h irradiation at 365 nm below 60 K, and (**d**) after annealing at 240 K. Insets in (**b-d**): (**b**) one dimer of precursor **1b**, (**c**) two adjacent carbene **2b**, and (**d**) one organometallic intermediate **3b**. Scanning parameters: (**b-d**) V_b = 100 mV, I_t = 10 pA; inset in (**b**) V_b = 100 mV, I_t = 5 pA, inset in (**c**) V_b = 100 mV, I_t = 10 pA, inset in (**d**) V_b = 10 mV, I_t = 50 pA.

For the formation of carbene **2b** on Ag(111), we photolyze precursor **1b** using 365 nm irradiation (Supplementary Fig. 12a), following the same procedure to form carbene **2a**. As we reported previously,[9] precursor **1b**, adsorbed at liquid nitrogen temperature, is homogeneously distributed as dimers across the Ag(111) surface (Supplementary Fig. 12b). Each diazo group forms a hydrogen bond to a vicinal hydrogen atom of its partner molecule in the dimer (Supplementary Fig. 12b, inset). Irradiation at 365 nm for 23.2 h converts each precursor **1b** to a species imaged as an ellipsoidal protrusion next to a depression (Supplementary Fig. 12c). The formed species were assigned to carbene **2b**, where the carbene draws electron density from the metal, leading to a reduced density of states in its vicinity.[9] Bowtie-shaped species are formed upon annealing at 240 K, which are imaged as two segments connected by a bright protrusion (Supplementary Fig. 12d). This species is assigned to an organometallic intermediate **3b**, with the bright protrusion an Ag adatom connecting two carbenes **2b** (Supplementary Fig. 12d, inset).

Supplementary Note 13: C−H activation of carbene 2b

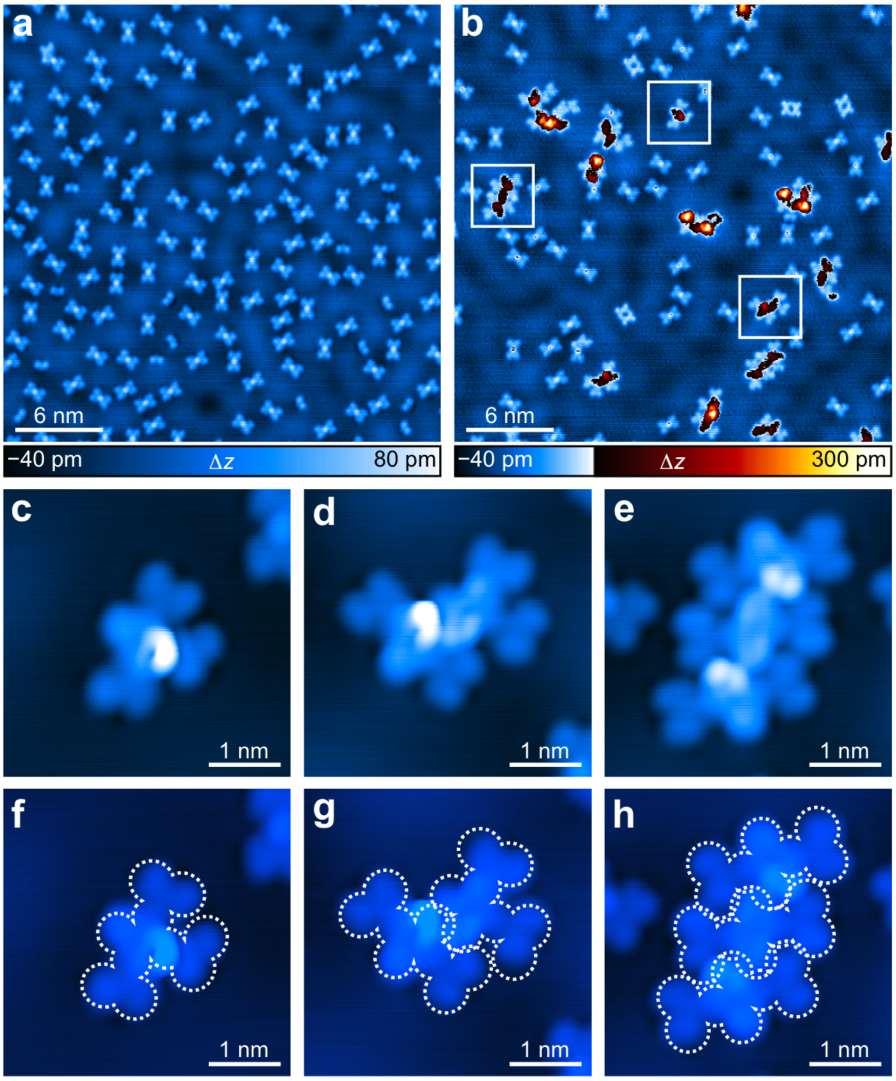

Supplementary Fig. 13. C−H activation of carbene 2b. a,**b**, Large-scale STM images of (**a**) carbene **2b** after annealing at 292 K and (**b**) after annealing at 375 K. **c-e**, Magnified images of the squares in (**b**). **f-h**, Images in (**c-e**) with white contours to guide the eye. Scanning parameters: (**a**) V_b = 100 mV, I_t = 5 pA, (**b**) V_b = 100 mV, I_t = 3 pA; (**c-e**) V_b = 10 mV, I_t = 5 pA.

Organometallic intermediates **3b** formed from carbenes **2b** remain stable at 292 K (Supplementary Fig. 13a). Some oligomers formed with bifluorenylidene (dimer **4b**) cores and fluorenyl branches at 375 K indicate that C−C coupling and C−H activation of carbene **2b** are both activated at this temperature (Supplementary Fig. 13b-h).

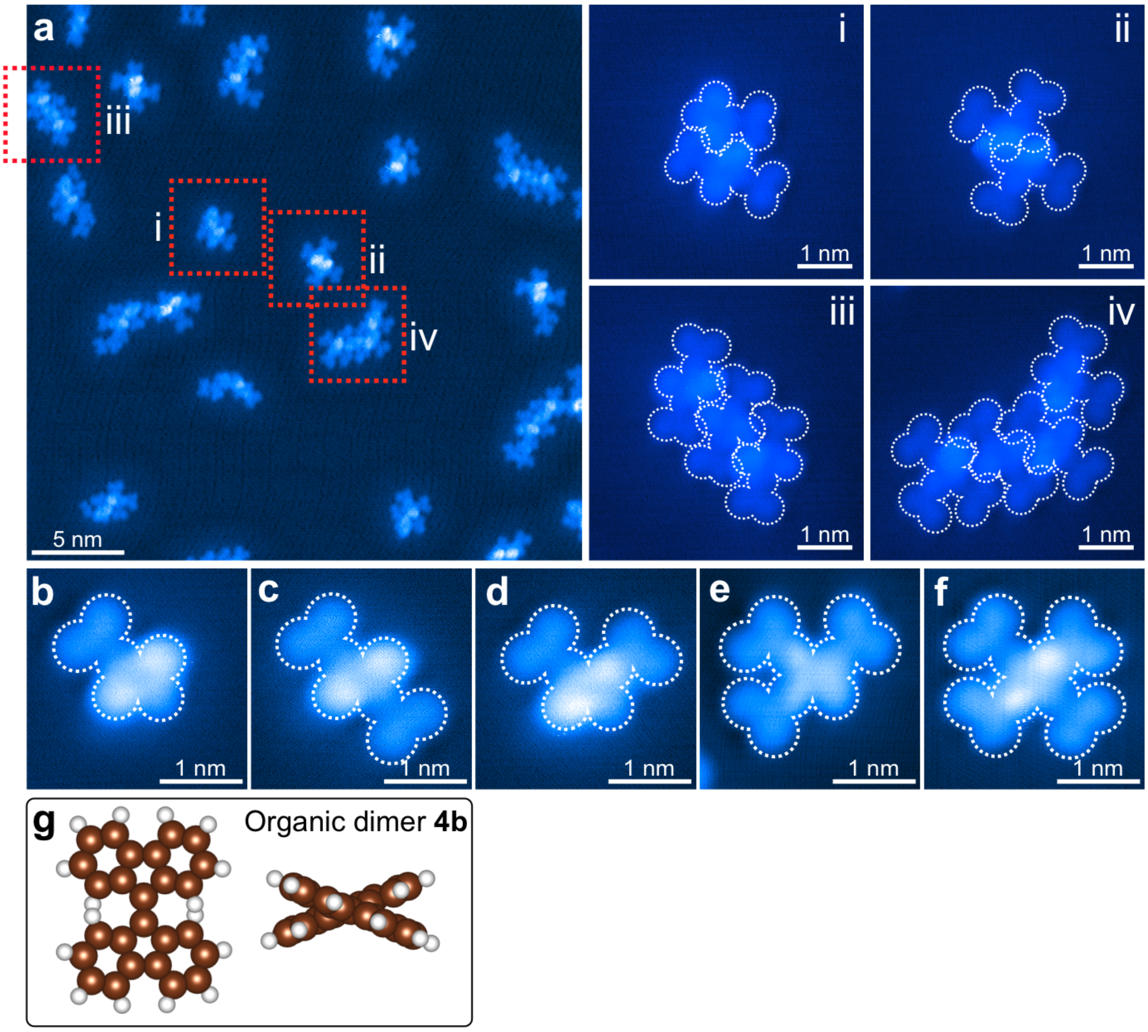

Supplementary Fig. 14. C−H activation of carbene 2b. a, STM images of carbene **2b** after annealing at 500 K with zooms of regions (i-iv). White contour lines are added to guide the eye. **b-f**, STM images of substituted dimers separated from their assemblies by lateral manipulation. **g**, Geometry of dimer **4b** (top and side view) in the gas phase (Data from NIST Standard Reference Database 69: NIST Chemistry WebBook). Scanning parameters: (**a-e**) V_b = 200 mV, I_t = 2 pA, (**f-h**) V_b = 300 mV, I_t = 2 pA, (**i**, **j**) V_b = 200 mV, I_t = 3 pA.

Annealing at 500 K converts all organometallic intermediates **3b** to oligomer agglomerates (Supplementary Fig. 14a). We identify the oligomers after lateral manipulation to separate these agglomerates. They consist of core structures with one to four branches (Supplementary Fig. 14b-f), similar to the substituted dimers formed via C−C coupling and C−H activation of carbene **2a** (Fig. 4b-g). The core structures exhibit two brighter and two less bright protrusions at opposite corners of a tetragon, consistent with a twisted geometry of dimer **4b** (Supplementary Fig. 14g).

Supplementary Note 14: Intramolecular cyclodehydrogenation

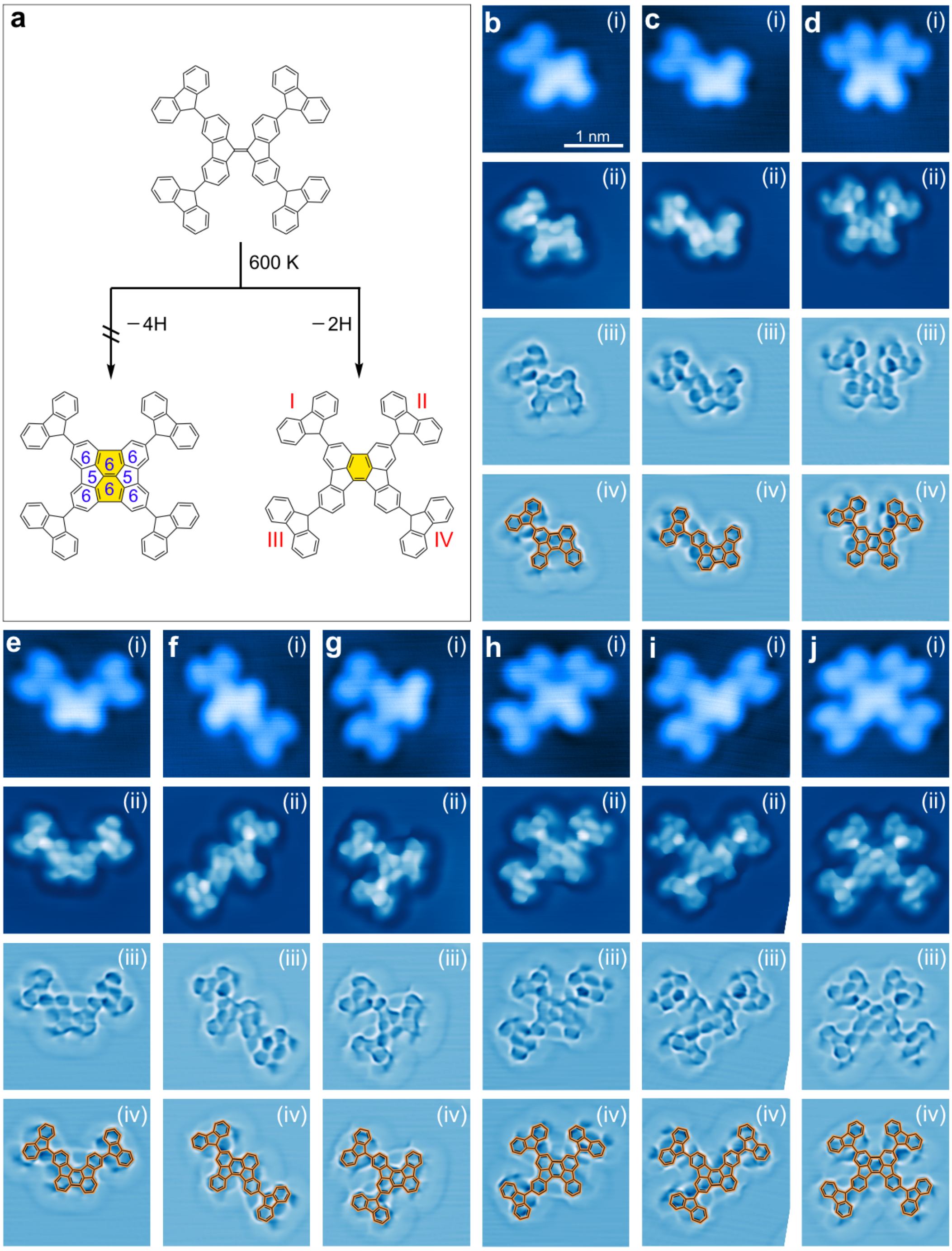

Supplementary Fig. 15. Intramolecular cyclodehydrogenation. a, Scheme of cyclo-dehydrogenation pathways. I, II, III, and IV mark the diaza-fluorenyl branches. **b-j**, STM images of the cyclodehydrogenation products formed at 600 K. (i): Images acquired with a metallic tip in constant-current mode; (ii): images acquired with a functionalized tip in constant-height mode; (iii): constant-height images processed with a Laplace filter. (iv): constant-height STM images superimposed with chemical drawings for orientation. All images use the same length scale. Scanning parameters: **b**(i)-**j**(i) V_b = 10 mV, I_t = 5 pA, **b**(ii) V_b = 10 mV, I_t = 500 pA, **c**(ii) V_b = 5 mV, I_t = 200 pA, **d**(ii)-**j**(ii) V_b = 5 mV, I_t = 300 pA.

In the main text, we discussed the intramolecular cyclodehydrogenation of substituted dimers with one to four branches (Fig. 5). Cyclodehydrogenation on either side generates a planar core structure in two different symmetries (Supplementary Fig. 15):

1. fused monosubstituted dimers: I (no mirror plane), III (no mirror plane)
2. fused disubstituted dimers: I-II (one mirror plane), III-IV (one mirror plane), I-IV (no mirror plane), I-III (no mirror plane)
3. fused trisubstituted dimers: I-II-III (no mirror plane), I-III-IV (no mirror plane)
4. fused tetrasubstituted dimer: I-II-III-IV (one mirror plane)

Cyclization at both sides would lead to a non-planar bowl-shaped structure (Supplementary Fig. 15a), similar to a fragment of a C_{60} molecule. Such a structure is energetically less favorable compared to the planar ones and is difficult to synthesize on surfaces[19]. In our system, it was not observed after annealing at 600 K.

Supplementary Note 15: Synthesis of chemical compounds

General materials and methods: Unless otherwise noted, all chemicals were used as commercially received. Benzene (p.a., VWR chemicals) was dried over 0.4 nm molecular sieves prior to use. NMR spectra were recorded on a Bruker AVIII-300 MHz spectrometer at room temperature, and the corresponding chemical shifts (δ) are given in ppm relative to tetramethylsilane.

Synthesis of 1,8-diaza-9-diazofluorene 1a.

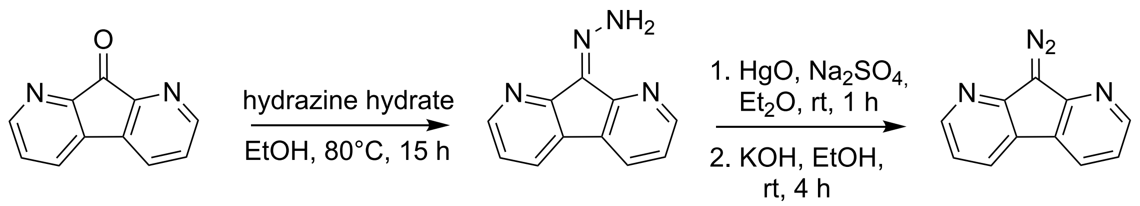

1,8-diaza-9-diazofluorene **1a** was synthesized in a two-step procedure[20,21]. First, 1,8-diazafluorenone hydrazone was synthesized from the reaction of 1,8-diazafluorenone with hydrazine hydrate (80%), and, second, the hydrazone was oxidized to the diazo compound. A mixture of dried hydrazone (0.19 g, 0.96 mmol), mercuric oxide (0.43 g, 1.97 mmol), and anhydrous sodium sulfate (0.24 g, 1.69 mmol) in dry Et_2O (20 mL) was stirred at room temperature under an argon atmosphere. After 1 h, a freshly prepared, concentrated solution of KOH in ethanol (0.3 mL) was added dropwise. The color of the solution changed from yellow to grey-black, and the solution was stirred for an additional 4 h. The resulting solution was filtered, washed with Et_2O, and concentrated to obtain 1,8-diaza-9-diazofluorene **1a** as orange crystals.

IR (Ar, 3 K): $\tilde{\nu}$ = 2096 (vs), 1585 (m), 1429 (s), 1401 (s), 1320 (m), 1254 (m), 1126 (m), 781 (m), 516 (m) cm^{-1}.

^{1}H NMR (300 MHz, DMSO-d_6): δ/ppm = 8.56 (dd, *J* = 4.9, 1.5 Hz, 2H), 8.49 (dd, *J* = 7.8, 1.6 Hz, 2H), 7.40 (dd, *J* = 7.8, 4.9 Hz, 2H).

^{13}C NMR (75 MHz, DMSO-d_6): δ/ppm = 151.03, 146.13, 129.60, 122.28, 119.99.

Synthesis of 9-diazofluorene 1b.

9-diazofluorene **1b** was synthesized according to literature procedures[22,23].

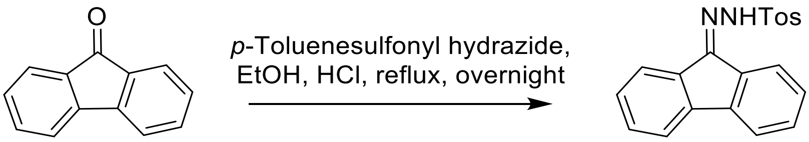

Step 1: 9-Fluorenone (2g, 11.1 mmol) and *p*-toluenesulfonyl hydrazide (4.26 g, 22.2 mmol) were suspended in 60 mL of ethanol. After the addition of 1 mL of concentrated HCl (aq.), the resulting solution was refluxed overnight. The mixture was cooled down to room temperature, and the resulting precipitate was filtered off, washed with small amounts of cold ethanol, and dried *in vacuo*. Further purification can be achieved by recrystallization from ethyl acetate. The synthesized 9-Fluorenone tosylhydrazone (2 g, 5.7 mmol, 52 % yield) was a yellow crystalline solid.

^{1}H NMR (300 MHz, DMSO-d$_6$): δ/ppm = 11.39 (s, 1H), 8.10 (d, *J* = 7.6 Hz, 1H), 7.93 (d, *J* = 8.3 Hz, 2H), 7.87 (d, *J* = 7.4 Hz, 1H), 7.83-7.78 (m, 1H), 7.61-7.50 (m, 2H), 7.48-7.40 (m, 4H), 7.32 (td, *J* = 7.5, 1.1 Hz, 1H), 2.39 (s, 3H).

^{13}C NMR (75 MHz, DMSO-d$_6$): δ/ppm = 151.60, 143.80, 141.47, 139.59, 135.80, 135.14, 131.85, 130.75, 129.40, 129.01. 128.34, 128.26, 127.91, 121.42, 120.68, 120.43, 21.06.

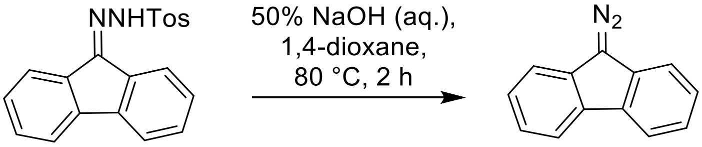

Step 2: 9-Fluorenone tosylhydrazone (2 g, 5.7 mmol) was dissolved in 25 mL of 1,4-dioxane. 5 mL of a 50% NaOH solution (aq.) was added, and the biphasic mixture was stirred strongly while being heated to 80 °C for 2 h. After cooling to room temperature, 20 mL of distilled water was added, and the mixture was extracted thrice with 30 mL diethyl ether. The combined organic phases were dried over sodium sulfate, and the solvent was evaporated *in vacuo*. The obtained microcrystalline orange powder was recrystallized from diethyl ether to yield 9-diazofluorene **1b** (827 mg, 4.3 mmol, 75% yield) as orange-red needles.

^{1}H NMR ($CDCl_3$, 300 MHz): δ/ppm = 8.09 (dt, *J* = 7.5, 1.0 Hz, 2H), 7.73 (dt, *J* = 7.7, 0.7 Hz, 2H), 7.41 (td, *J* = 7.5, 1.3 Hz, 2H), 7.34 (td, *J* = 7.4, 1.2 Hz, 2H).

^{13}C NMR (75 MHz, DMSO-d$_6$): δ/ppm = 132.45, 130.67, 126.40, 124.47, 121.10, 119.76.

Synthesis of dimer 4a.

Dimer **4a** was synthesized in three steps according to literature procedures[20,24].

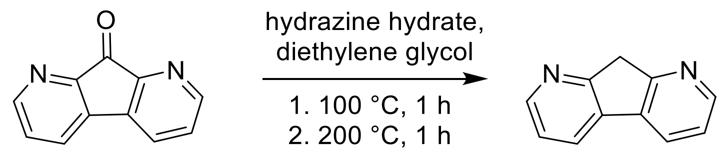

Step 1: 1,8-Diazafluoren-9-one (0.5 g, 2.7 mmol) was suspended in diethylene glycol (4 mL) and hydrazine hydrate (1 mL, 80%). The solution was heated at 100 °C for 1 h. Then, the temperature was increased to 200 °C for 1 h. After cooling down to room temperature, it was poured into water (50 mL), and the aqueous phase was extracted twice with chloroform (20 mL). The combined organic phases were washed with water, dried over sodium sulfate, and the solvent was removed *in vacuo*. The obtained brown-pinkish solid was either already sufficiently pure for the next step or it was further purified by flash column chromatography (silica gel, ethyl acetate/methanol 9:1). The synthesized 1,8-diazafluorene (250 mg, 1.5 mmol, 56% yield) was as a pinkish solid.

^{1}H NMR (300 MHz, $CDCl_3$): δ/ppm = 8.56 (dd, *J* = 5.0, 1.6 Hz, 2H), 8.01 (dd, *J* = 7.7, 1.6 Hz, 2H), 7.32 (ddt, *J* = 7.8, 5.0, 0.8 Hz, 2H), 4.13 (s, 2 H).
^{13}C NMR (75 MHz, $CDCl_3$): δ/ppm = 162.69, 148.70, 133.26, 128.03, 122.23, 40.69.

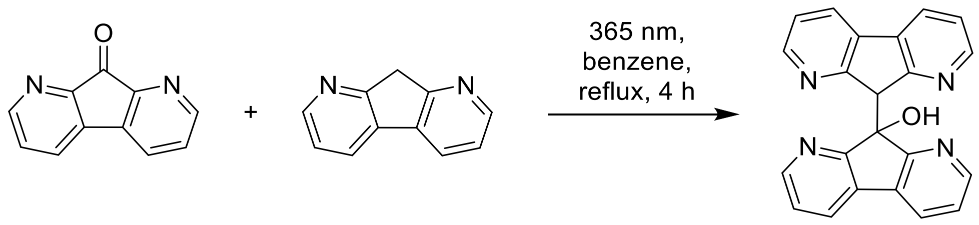

Step 2: A mixture of 1,8-diazafluorene (250 mg, 1.5 mmol) and 1,8-diazafluoren-9-one (270 mg, 1.5 mmol) was suspended in dry benzene (25 mL). The solution was heated to reflux and irradiated for 4 h with a 365 nm LED (5 W) while maintaining the temperature. Upon ending the irradiation, the solution was cooled down to room temperature, and the solvent was removed *in vacuo*. The remaining solid was dissolved in hot acetone, and the pure product separated as fine white powder upon cooling down to −20 °C in a freezer overnight. The white powder was filtered off and washed with small amounts of cold acetone. The mother liquid was concentrated *in vacuo*, and this crystallization procedure

was repeated several times until the precipitate showed a slight brown-reddish coloration, indicating leftover starting material. 1,1',8,8'-tetraazabifluoren-9-ol (150 mg, 0.4 mmol, 29% yield) was obtained as a white solid.

^{1}H NMR (300 MHz, $CDCl_3$): δ/ppm = 8.32 (s (very broad), 4H), 7.86 (d, *J* = 7.4 Hz, 2H), 7.72 (d, *J* = 7.6 Hz, 2H), 7.61 (s, 1H), 7.22 (d (very broad), 2H), 5.13 (s, 1H).
^{13}C NMR (75 MHz, $CDCl_3$): δ/ppm = 164.21, 151.41, 148.96, 148.70, 148.04, 133.11, 132.38, 128.72, 128.04, 127.85, 127.53, 123.65, 122.68, 122.23, 81.33, 52.88.

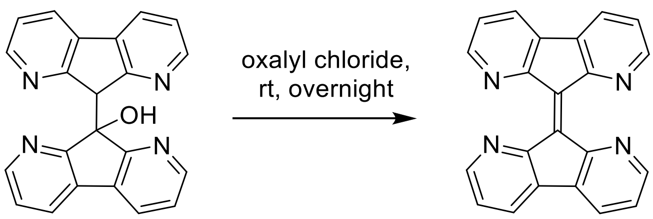

Step 3: Oxalyl chloride (0.7 mL, 8.2 mmol) was added to 1,1',8,8'-tetraazabifluoren-9-ol (150 mg, 0.43 mmol) under stirring. The white powder immediately turned red-brown upon addition, and stirring was continued overnight at room temperature. A saturated aqueous sodium carbonate solution was added, and the aqueous phase was extracted several times with chloroform (~1.5 L total). The combined organic phases were dried over magnesium sulfate, and the solvent was evaporated *in vacuo*. The product, 1,1',8,8'-tetraazabifluorenylidene (dimer **4a**), was obtained as a deep red solid (120 mg, 0.36 mmol, 84% yield).

^{1}H NMR (300 MHz, $CDCl_3$): δ/ppm = 8.65 (dd, *J* = 4.9, 1.6 Hz, 4 H), 7.92 (dd, *J* = 7.7, 1.6 Hz, 4H), 7.25 (dd, *J* = 7.3, 5.3 Hz, > 4H*).

* Partial overlap of signal with solvent signal influences observed integral.

Note: measurement of a ^{13}C NMR spectrum of **4a** was not possible due to the low solubility of the compound in several typical NMR solvents.

Supplementary References

1. A. H. Raut, PhD thesis, Ruhr Universität Bochum, 2019.
2. Hoffmann F. M. Infrared reflection-absorption spectroscopy of adsorbed molecules. *Surf. Sci. Rep.* **3**, 107-192 (1983).
3. Artyushkova K. Misconceptions in interpretation of nitrogen chemistry from X-ray photoelectron spectra. *J. Vac. Sci. Technol. A* **38**, 031002 (2020).
4. Cao Y. et al. C−C coupling of carbene molecules on a metal surface in the presence of water. *J. Am. Chem. Soc.* **145**, 11544-11552 (2023).
5. Rao R. M., Beuhler R. J. & White M. G. Nonthermal photodesorption of N_2 from Ag(111). *J. Chem. Phys.* **109**, 8016-8026 (1998).
6. Bourissou D., Guerret O., Gabbai F. P. & Bertrand G. Stable carbenes. *Chem. Rev.* **100**, 39-92 (2000).
7. Li Y. Z. & Schuster G. B. Photochemistry of 9-diazo-3,6-diazafluorene: through-space or through-bond transmission of electronic effects. *J. Org. Chem.* **52**, 3975-3979 (1987).
8. Zhukhovitskiy A. V., MacLeod M. J. & Johnson J. A. Carbene ligands in surface chemistry: from stabilization of discrete elemental allotropes to modification of nanoscale and bulk substrates. *Chem. Rev.* **115**, 11503-11532 (2015).
9. Mieres-Perez J. et al. Controlling reactivity − real-space imaging of a surface metal carbene. *J. Am. Chem. Soc.* **143**, 4653-4660 (2021).
10. Lucht K. et al. On-surface alcohol formation from a carbene precursor. *J. Phys. Chem. C* **128**, 15347-15355 (2024).
11. Gawronski H., Carrasco J., Michaelides A. & Morgenstern K. Manipulation and control of hydrogen bond dynamics in absorbed ice nanoclusters. *Phys. Rev. Lett.* **101**, 136102 (2008).
12. Hapala P. et al. Mechanism of high-resolution STM/AFM imaging with functionalized tips. *Phys. Rev. B* **90**, 085421 (2014).
13. Hamalainen S. K. et al. Intermolecular contrast in atomic force microscopy images without intermolecular bonds. *Phys. Rev. Lett.* **113**, 186102 (2014).
14. Díaz-Requejo M. M. & Pérez P. J. Coinage metal catalyzed C-H bond functionalization of hydrocarbons. *Chem. Rev.* **108**, 3379-3394 (2008).
15. Lawrence J. et al. Topological design and synthesis of high-spin aza-triangulenes without Jahn-

Teller distortions. *ACS Nano* **17**, 20237-20245 (2023).

16. Zhao Y. et al. Quantum nanomagnets in on-surface metal-free porphyrin chains. *Nat. Chem.* **15**, 53-60 (2023).
17. Pavlicek N. et al. Synthesis and characterization of triangulene. *Nat. Nanotech.* **12**, 308-311 (2017).
18. Morgenstern K., Lorente N. & Rieder K. H. Controlled manipulation of single atoms and small molecules using the scanning tunnelling microscope. *Phys. Status Solidi B* **250**, 1671-1751 (2013).
19. Wang T. et al. Challenges in the synthesis of corannulene-based non-planar nanographenes on Au(111) surfaces. *Phys. Chem. Chem. Phys.* **23**, 10845-10851 (2021).
20. Schönberg A. & Junghans K. Versuch mit 1,8-Diaza-Fluoren-Derivaten und über Tetraaryläthylene mit "anomalem" Verhalten. *Chem. Ber.* **95**, 2137-2143 (1962).
21. Levy A., Cohen S. & Agranat I. Overcrowded 1,8-diazafluorenylidene-chalcoxanthenes. Introducing nitrogens at the fjord regions of bistricyclic aromatic enes. *Org. Biomol. Chem.* **1**, 2755-2763 (2003).
22. Jonczyk A. & Wlostowska J. A simple method for generation of diazocompounds in an aqueous two-phase system. *Synth. Commun.* **8**, 569-572 (1978).
23. Feng X. W. et al. Copper-catalyzed nitrogen loss of sulfonylhydrazones: a reductive strategy for the synthesis of sulfones from carbonyl compounds. *Org. Lett.* **12**, 4408-4411 (2010).
24. Alan E. R., John M. M. & Johannes N. V., inventors; Alpha, Alpha-disubstituted aromatics and heteroaromatics as cognition enhancers patent EP311010A2. 1989.
